# Recruiting cancer survivors into research studies using online methods: a secondary analysis from an international cancer survivorship cohort study

**DOI:** 10.3332/ecancer.2019.990

**Published:** 2019-12-12

**Authors:** Nicholas J Hulbert-Williams, Rosina Pendrous, Lee Hulbert-Williams, Brooke Swash

**Affiliations:** Centre for Contextual Behavioural Science, School of Psychology, University of Chester, Chester CH1 4BJ, UK; a http://orcid.org/0000-0001-9041-5485; b http://orcid.org/0000-0002-9085-9876; c http://orcid.org/0000-0001-5892-6488; d http://orcid.org/0000-0003-2457-954X

**Keywords:** survivorship, online, recruitment, methodology, psychosocial

## Abstract

Recruiting participants into cancer survivorship research remains a significant challenge. Few studies have tested and compared the relative use of non-clinical online recruitment methods, especially in samples of adult cancer survivors. This paper reports on the feasibility of recruiting a representative cohort of cancer survivors using online social media. Two hundred participants with cancer diagnosis within the past 12 months were recruited via social media (Facebook, Twitter and Reddit) into a longitudinal questionnaire study. Different methods of online recruitment proved to be more effective than others over time. Paid Facebook boosting, Reddit posts and Twitter advertisements placed by existing cancer charities proved most helpful in reaching our recruitment target (contributing 27%, 22% and 32%, respectively). Recruiting online achieved a more demographically and clinically representative sample for our study: our subject was younger, less heteronormative, including those with a range of clinical diagnoses, primary and recurrence illness, and patients who had both completed and were still receiving treatment. This was certainly not a quick method of sample recruitment but that could have been optimised by focussing only on the three most effective methods described earlier. While we found that online recruitment is significantly lower in cost than traditional recruitment methods, and can reduce some biases, there still remains the potential for some biases (e.g. excluding much older participants) and ethical/methodological issues (e.g. excluding those without access to Internet). We outline our recruitment strategy, retention rates and a cost breakdown in order to guide other researchers considering such methods for future research in cancer survivorship.

## Background

Recruiting patients into cancer survivorship research presents a significant challenge, and yet continued research on this growing population is necessary, in particular, to address psychological supportive care needs [1]. There remains a lack of awareness of the barriers to recruitment in this population. Failing to address these barriers may increase the risk of publication bias [2], particularly in randomised clinical trials [3]. Psychosocial oncology research often tends towards quantitative and makes heavy use of inferential statistics, the primary purpose of which ‘…is to draw inferences about parameters (characteristics of populations) from statistics (characteristics of samples)’ [4]. The processes by which participants are recruited are the pillars upon which rests the external validity of research studies. However, the more recruited samples differ from the population of interest, the less confidence we can have in the external validity or generalisability of the findings. In cancer survivorship research, such threats to validity are particularly acute.

### Why is recruitment so challenging?

Barriers to recruitment in health research can include: resources (mainly cost and funding restrictions), patient ineligibility (e.g. cancer stage, comorbidities and risk of death), failure to participate due to poor or decreasing health, and preference for specific treatments and patient characteristics [2, 5]. Language barriers, infrastructure (e.g. additional hospital visits/travel time) and participant characteristics (especially altruism) may also be important predictors of willingness to participate in research [6], in both patients and caregivers [3]. Emphasising the benefits of research participation, using information technology to create easier routes into participation for those with access issues [6] and allowing self-referral into research [7], may improve recruitment rates and provide information about demand and patient characteristics.

Recruitment rates into trials of interventions are often higher than observational or questionnaire-based studies as the benefits of participation (receiving an intervention) are more explicit. However, longitudinal questionnaire studies have an important place in survivorship research by modelling which variables—demographic, clinical and psychosocial—predict changes in well-being over time. This can be useful for clinical monitoring and risk stratification [8] and to identify potential statistical moderators of patient-reported outcomes [1, 9], thus informing data-driven intervention design. Recruiting into these studies is typically even more challenging [10]; the perceived burden is high and individual benefit is less clear.

Survey studies in cancer survivorship most typically recruit from the clinical setting. Within the UK, however, this is becoming increasingly difficult. Putting aside problems of clinical gatekeeping [6, 11], increasingly limited healthcare resources may lead to de-prioritisation of research promotion. One potential solution, implemented in the UK in 2004, was the development of the UK Clinical Research Network (UKCRN) which has resulted in better quality and regulated research [12]. Research that is adopted onto the UKCRN Portfolio can use a dedicated team of Research Nurses based usually in the clinical setting to help with recruitment [13]. However, this increases the cost of survivorship research and limits access to only those studies eligible for portfolio adoption; research funded by non-National Institute for Health Research (NIHR)-affiliated charities and universities and the majority of postgraduate research are excluded. Of those adopted, priority is given to treatment trials over survivorship research: only 2.1% of patients who entered into UK clinical cancer trials between 2012 and 2014 were recruited to psychosocial oncology and survivorship research [14]. Alternative recruitment methods have included platforms such as local radio, postal invitation or newspaper advertisement with varying success.

### Is online recruitment a potential solution?

There is a growing interest in the use of online social media in research [15], but currently, this is rare in cancer survivorship. This may be a demographic issue that will resolve over time. Compared to other research where online recruitment is common, the cancer survivor population is older [16]; however, social media use is increasing in older populations [17] and 71% of the 55–75 year age group in the UK now own a smartphone [18]. In one acceptability study, 79% of childhood cancer survivors (18–48 years of age) reported positive attitudes towards the use of social media in research study recruitment, with 80% reporting at least weekly use of social media [19]. As cultures become increasingly digital, these methods of recruitment become more possible in cancer survivorship research.

Facebook currently has 2.23 billion users worldwide [20] and provides a platform to post new content to specific groups or pay to advertise to the whole Facebook community. Whitaker *et al* [21] systematically reviewed the use of Facebook for healthcare research recruitment concluding several benefits: reduced cost, quicker recruitment and more representative samples, particularly for hard to reach demographics. Only one of the included studies was in cancer (recruiting parents of children with cancer) [22]. In 10.5 weeks, the advertisement generated 3,897,981 impressions (views) and 1,050 clicks, with an average cost-per-click estimated at $1.08. Out of 300, 45 people went on to view the survey fully participated; at a total recruitment cost of $1129.88, this study demonstrated the potential cost-effectiveness of this recruitment method for this population. Elsewhere, there are cautions: self-screening methods assume participant honesty, and recruitment rates in some studies are as low as 1% because advertisements are shown to millions of Facebook users worldwide [23].

Reddit is a free, forum-based platform, which tends towards more interactive discussion among community users than Facebook. Organised into a number of different discussion forums (‘sub-reddits’) and organised around topic areas, posts can be commented on (as with Facebook) and can be ‘up-’ or ‘down-voted’ by group members as a way of indicating post priority (similar to a Facebook ‘like’). Data from January 2019 indicated that 1.65 billion people had used Reddit in the preceding 12 months [24]. Reddit may enable the targeting of more specific populations than Facebook [25]. To our knowledge, no studies have reported on the efficacy of Reddit as an online source for recruiting cancer survivors.

There are on average 321 million monthly active Twitter users worldwide [26]; taking just three UK-based cancer charities as examples, Macmillan Cancer Supports has 674,200 followers, Breast Cancer Care has 159,800 followers and the Teenage Cancer Trust has 105,600 followers. There are just two studies reporting on the use of Twitter for recruitment into cancer survivorship research. Rabin *et al* [27] reached 11 potential participants through social media (no data were provided on comparisons between Twitter and other platforms) though none were actually eligible to participate, highlighting potential self-screening issues. Keaver *et al* [28] recruited through Twitter for a cross-sectional study on willingness to participate in a nutrition and web-based intervention research. They concluded that while Twitter is a feasible recruitment method, samples might be biased towards younger, female, more educated and less ethnically diverse participants.

## Study aim

This paper reports on a secondary analysis of data collected in a longitudinal study exploring predictors of patient-reported outcomes in cancer survivors over a 2-year period: this was an early phase study to inform the development of tailored Acceptance and Commitment Therapy (ACT)-based interventions [29] for cancer survivors based on our previous conceptual and pilot cross-sectional research [30–32]. The main study is still ongoing. As a secondary aim of this study, we trialled the feasibility of recruiting cancer survivors through online recruitment, comparing the relative use of different social media platforms. These secondary analyses are reported and discussed in this paper.

## Method

Ethical approval for this study was provided by the University of Chester, UK (Ref: 2001316) and the University of Sydney, Australia (Ref: 2016/752).

## Design

The study from which these recruitment data are drawn uses a longitudinal, cohort design. Participants complete online, self-report questionnaires at baseline, and then at 3-month intervals thereafter, for up to 2 years. One UK-based participant requested a traditional paper-and-post questionnaire which was sent at equivalent time points to the online sample. Participants are entered into a prize draw to win a £50 Amazon shopping voucher at each time point as an incentive; those who complete all nine questionnaires will be entered into a further prize draw to win an iPad Mini. At each time point, participants completed a batch of repeated-measures questionnaires, including the revised Acceptance and Action Questionnaire (AAQ-II) [33]; the Brief Experiential Avoidance Questionnaire (BEAQ) [34]; the Cognitive Fusion Questionnaire (CFQ) [35]; the Mindful Awareness and Attention Scale (MAAS) [36]; the Engaged Living Scale (ELS) [37]; the short Depression, Anxiety and Stress Scale (DASS-21) [38]; the EQ-5D [39]; the Assessment of Survivor Concerns Scales (ASC) [40]; and a revised version of the Benefit Finding for Breast Cancer Scale [41], with reference to breast cancer omitted for broader applicability (as previously published) [31]. Participants also completed a demographic and clinical questionnaire at baseline and were asked to report on changes to the clinical nature of their cancer and/or treatment at each follow-up phase. To reduce participant burden, we selected the shortest, psychometrically sound scales for each variable available at the time of study initiation. In total, questionnaire length was 112 items at baseline, and 100 items at follow-up. Completion was estimated to take between 30 and 45 minutes, dependent on reading speed. Online questionnaires were administered using the LimeSurvey platform, which presents the questions in the form of a webpage which formats itself appropriately for screen and device type.

## Participants: eligibility and target sample size

Participants confirm that they met the following eligibility criteria at consent.

- Over the age of 16 years at the time of consent.
- Received a cancer diagnosis (including recurrence) within the past 12 months.
- Good comprehension of written English (translation into other languages was not possible within our study budget).

As our recruitment design was relatively novel, there was very little on which to base an estimated likely response rate. Our sample size calculation was based on the assumption that between 10% and 15% of participants would be lost to attrition at each follow-up stage. We thus aimed to recruit in excess of 500 participants, to ensure a final follow-up sample of at least 150 participants to adequately power multivariate regression modelling [42].

## Procedure

Study advertisements were distributed through a variety of online, social media platforms. Where necessary, permission was granted by the moderators or administrators beforehand. Advertisements contained a web-link to a longer study invitation letter which then linked to the online participant information sheet, followed by an online consent form, and then the questionnaire itself.

**Figure 1.**
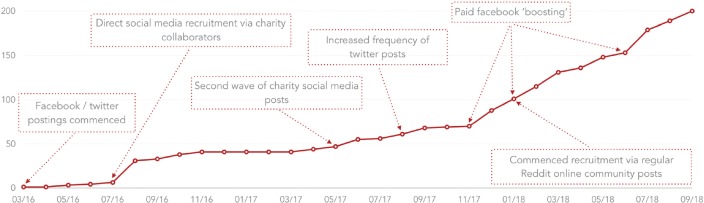
Cumulative recruitment and notable changes in recruitment strategy over time.

Our initial recruitment took place from March (month 1) to November 2016 (month 7), with study advertisements placed on a dedicated Twitter account and Facebook page (see Figure 1). Throughout this period, a member of our research team actively used those accounts to ‘retweet’ and ‘like’ content provided by other social media users to boost the number of followers to those study-specific accounts, as a means to maximise reach through ‘retweets’ and ‘likes’ of our own content. During month 6, a number of UK cancer charities retweeted posts and placed dedicated advertisements on their own Facebook pages.

After a short break to review our strategy, we recommenced recruitment in April 2017 (month 13) for a further 18 months through to September 2018 (month 30). We continued advertisements from our own dedicated Twitter and Facebook accounts, though with a greater emphasis on members of the research team retweeting and sharing (via Facebook) to maximise circulation within personal social media networks. We requested two further waves of charity retweeting. In months 20, 22 and 27, we paid for Facebook ‘boosts’. We began posting advertisements to Reddit online community groups from month 21, continuing for 9 months until the close of recruitment in August 2018. Follow-up data collection will continue until September 2020.

Over a 3-month period in Spring 2018, we attempted to supplement recruitment using a local distribution newspaper advertisement (total readership estimate: 10,000 readers across one single print-copy and associated online presence) and by attending local community interest (four in total) and cancer support groups (six in total). We were able to analyse recruitment rates from these sources separately because participants were required to contact the research team directly to request a link to the survey website.

## Data analysis

We analyse data on online recruitment feasibility according to three key metrics.

- Comparative speed and success of recruiting cancer survivors through Twitter, Facebook and Reddit.
- Representativeness of the recruited sample.
- Cost-considerations of employing online recruitment methods.

Our results are analysed and presented descriptively, given the scope of these study aims.

## Results

### Overview

Two hundred ninety-four individuals consented and accessed the questionnaire, but only 202 submitted a complete data set (68.7% completion rate). Two were excluded as they did not meet the study inclusion criteria and 19 (8% of the recruited sample) did not provide valid e-mail addresses for follow-up. The majority were recruited online, with just one participant recruited through the newspaper advertisement and one recruited through a community group (1% of our total sample collectively). No participants were recruited through local cancer support groups; with few exceptions, this was because most of the support group attendees exceeded the time-since-diagnosis eligibility criteria. Forty-one participants were recruited across the first wave of recruitment; at least 28 of these coincided with a retweet or a post from a charity collaborator highlighting the importance of partnering with other online organisations with an established social media following. The most effective sources of recruitment over the second wave of recruitment were paid Facebook ‘boosts’ and Reddit posts (see Figure 1).

## Speed and success of recruitment through various social media platforms

### Twitter

During the first 5 months of recruitment, six participants were recruited through posts from our study-specific account. This recruitment rate was replicated at other times where no other specific recruitment activities were taking place, providing a baseline of one participant recruited per month (23 in total). During recruitment month 15, we increased the tweet frequency per week, contributing six additional participants through recruitment (14.5% of the total sample recruited through direct Twitter advertisements). During months 6–9, 18–20 and 29, we engaged with cancer charities through Twitter, encouraging them to retweet our advertisement or to directly tweet a study invitation. This was more successful during the first time-period, but in total, we estimate that charity engagement online resulted in the recruitment of 64 participants (32.0% of our total sample).

We undertook two detailed audits of Twitter activity, first in January 2018, and again updated in March 2019. Each Twitter advertisement was viewed a median number of 313 times (range = 19–34,084; mean = 904), with between 0 and 86 people clicking the embedded web-link per tweet (‘clicks’: median = 1; mean = 4.15). These data are not appropriate for inferential statistical analysis, but our audit suggests that tweets placed between 23:00 and 09:00 hours Greenwich Mean Time (GMT) were most effectively distributed; our most successful tweet—which was viewed 34,084 times and was retweeted 65 times—was placed at 01:02 hours GMT. While these tentative findings might not generalise, it is probable that timing is an important consideration for online recruitment. It is impossible for us to calculate a definitive number of independent Twitter users reached, given an overlap between followers of the various retweeting accounts and fluctuations in Twitter account followings over time.

### Facebook

At the time of writing this manuscript (April 2019), our study-specific Facebook group had 859 subscribed followers though this has increased slowly through the recruitment period. We were cautious not to post too frequently so as not to appear that we were ‘spamming’ followers’ timelines. Six free-of-charge study advertisements were posted during the first 18 months of recruitment: collectively, these attracted eight ‘likes’, 13 ‘shares’ and seven ‘comments’, indicating low-level engagement. We do not believe that any of our recruited samples were recruited through these posts. During month 22, free-of-charge advertisements were placed directly on 17 other cancer-specific Facebook pages; this was more successful, resulting in seven participants recruited (3.5% of the total sample).

Facebook allows users to pay for post ‘boosting’ which prioritises posts to targeted users online feeds. We paid for three separate ‘boosted’ recruitment advertisements in total, targeting both male and female users, over 16 years of age, concentrated on the following geographical locations: England, Scotland, Wales, Northern Ireland, Canada, Australia and New Zealand. Each ‘boost’ was designed to spread the advertisement over seven days. These ‘boosted’ advertisements reached 10,623, 13,142 and 204,609, respectively (‘reach’ is defined by Facebook as the number of unique users to whom the advertisement was targeted), making 185,724 impressions (advertisement views or reads), and recorded 120,445 direct engagements (active ‘clicks’, ‘likes’, ‘shares’ or ‘comments’). Despite increased engagement, there was continued passivity of interaction–most of the ‘comments’ focused on disclosure of diagnosis or the seeking of peer support, rather than comments about the study specifically. We estimate that 54 participants (27.0% of the total sample) were recruited through this boosted Facebook advertising.

### Reddit

During the final 8 months of recruitment, we advertised on 10 cancer-specific sub-reddits, five health-related sub-reddits and two research-participation oriented sub-reddits. Membership of these sub-reddits ranged from just 10 (‘psychooncology’ sub-reddit) to 563,000 (‘health’ sub-reddit), with an average of 73,816 members per group. We posted to each between 1 and 10 times dependent on engagement to early postings. Forty-nine advertisements were placed in total, which were up-voted between 0 and 15 times; 10 were commented on by community members showing higher engagement. We estimate that 44 participants were recruited through Reddit posts (22% of the total sample) but over a considerably shorter period of time than other methods.

## Representativeness of the recruited sample

Table 1 (below) summarises the demographic and clinical characteristics of our sample. As we would often see in cancer survivorship research, there was a female bias, though the mean age is slightly younger, and the range greater, than we might otherwise expect. Wakefield *et al* [43], for example, report that across 155 international surveys of adult cancer patients, the overall mean age of participants is 53.39 years (SD = 14.5; range = 24–64), though in a recent survey of UK cancer survivors recruited through clinical services (with similar inclusion criteria and aims to the current study) we recruited a sample with a mean age of 61.4 years (SD = 16.8, range = 32.90) [31]. The proportion of participants disclosing as non-heterosexual is higher than we often see in this kind of research study; a recent secondary analysis of the UK Cancer Patient Experience Survey (which recruited through the NHS), for example, reported that less than 1% identified as lesbian, gay or bisexual [44]. The majority of our participants were recruited from the UK, which is not surprising as we recruited for longer in this country, and we partnered with a greater proportion of UK charities than those in other countries. We were able to recruit cancer survivors from 12 countries in total, including those less represented in the literature: for example, Turkey, Central and South America, The Philippines and South Africa.

Clinically, we recruited cancer survivors of those diagnoses corresponding to the focus of charities that were more willing to engage with our study; as a result, we have a breast cancer bias, though this is far reduced in comparison to other published research with mixed diagnosis samples. We were able to recruit a good mix of participants who had received both primary and recurrent diagnoses and who, at the time of consent, were still undergoing or had completed their active cancer treatments.

## Cost considerations

Recruiting participants into this study has taken approximately 1 hour per week for the 30-month duration. This includes the placement of all online materials, regular monitoring of (and responding to) comments and interactions, liaising with charity partners, and managing the recruitment queries email address. A significant portion of time (approximately 40 hours) was spent over a 2-month period where we attempted, somewhat unsuccessfully, face-to-face recruitment at community and cancer support groups. Given that the researcher undertaking recruitment activities was doing much of this activity either virtually (online) or in general population samples rather than in clinical settings, it was possible to appoint a more junior member of staff with less relevant cancer-related expertise. Excluding study-set up activities (e.g. ethical approvals) and costs associated with data collection, the direct staffing costs of this recruitment activity equate to approximately £2000. This is substantially cheaper than clinic-based recruitment which often necessitates: 1) higher grade and more experienced research staff; 2) travel costs and unproductive time waiting for referrals in clinics and 3) in some cases in the UK, additional Research Support costs to access the UKCRN. To formulate a cost-comparison for just this latter point, we modelled Research Support costs for recruitment had we used the UKCRN; allocating time for study set-up meetings and clinical staff briefings (90 minutes), regular study monitoring meetings with recruiting nurse teams (60 minutes, every 3 months, for 30 months), eligibility screening (1 hour per week for 30 months), and study introduction/informed consent meetings with each participant (*n* = 200), this cost alone would be £9,708 (calculation correct as of August 2019), and that would be in addition to staff employed at the university to undertake all other research-related activities. Online recruitment also enabled a more time-flexible working pattern which would not have been possible had the research been recruiting directly from clinics.

Regarding other recruitment costs, we paid a total of £625 for Facebook ‘boosting’, £380 for the newspaper advertisement, and approximately £50 for travel and printing costs related to the face-to-face recruitment attempts. If we exclude the latter two of these, the combination of Facebook charges and staffing required to recruit the 198 participants recruited through electronic methods equates to a cost of £13.25 per participant recruited.

Moving forward with our study, 199 of our participants (99.5%) have agreed to complete our surveys using electronic methods, which enables further cost-savings. LimeSurvey is an open-source software which can be installed on an institution’s web-server for free. In most cases, the only costs associated with data collection are, again, time of survey administration. This could be further reduced by using a survey distribution platform that enables automated follow-up administration, but this was not possible in our case. Nonetheless, the cost-saving of using online questionnaires can be favourably compared with the £15 printing and postage costs (not to mention staff time for data input) for the participant completing the questionnaire through paper means. Had our full sample participated in a paper and post format, this would have added a further £3000 to our study cost. Online data collection also has added benefits for the environment, of course.

## Discussion

Longitudinal ‘cohort’ studies of adjustment to cancer survivorship are a crucial part of the ongoing psychosocial oncology and supportive cancer care research effort. They help researchers and clinical teams alike to better understand the prevalence of long-term consequences of cancer treatment [8], including the enduring psychological impact [45, 46], and they provide important data to build effective interventions to improve survivorship care [1, 9]. While there are some excellent examples of cohort studies of cancer survivors [46–49], ours is the first to explore third-wave, ACT-based, psychological processes as predictors of patient-reported psychosocial outcomes. Many of these other cohort studies represent incredibly expensive and logistically complex research projects. The CREW Cohort Study, for example, recruited nationally using UK CRN nurses across 30 UK cancer centres [50] for 17 months to achieve their sample of 1056 colorectal cancer survivors [51]. As detailed in our background section to this paper, the high cost of these traditional recruitment methods limits the number of these types of studies that can be undertaken.

A secondary aim of our research, as reported in this paper, was to explore whether a sample of cancer survivors could be recruited using more cost-effective, online methods. We also aimed to explore whether these methods might enable us to recruit a demographically and clinical representative sample to improve on some of the age, gender, sexuality and clinical biases commonly reported in cancer survivorship research [2, 31, 43, 44, 52–56].

Regarding recruitment feasibility, our analysis focused on the speed and success of recruitment, and the cost-considerations of online recruitment methods. After a period of 30 months, we closed recruitment with a total sample size considerably smaller than we had anticipated. Our decision to close recruitment was made pragmatically because of the time that recruitment had already taken. The data from this study highlight particularly three effective methods of online recruitment (established charity Twitter posts, Facebook boosts and Reddit posts); were we to have had this knowledge prior to this study, and implemented these methods from the start of recruitment, we believe we would have reached our full sample in approximately the same length of time (estimated 16 participant recruits per month for 31 months).

One important limitation from our study is that we did not include a question on the survey about where the participant was recruited from. This means that: 1) we cannot compare sample characteristics between different social media platforms and 2) we cannot be absolutely certain how each participant was recruited. We are, however, able to make inferences about where participants were recruited from in various months of the study, given that we altered focus throughout the study and kept systematic records of differential responses per month following a shift in recruitment focus. A future study which better captures these data would be a helpful methodological contribution to the cancer survivorship literature.

Comparing different online recruitment methods, there were distinct limitations to using both Twitter and Facebook on an *ad hoc* basis, from study-specific platform accounts: recruitment, we conclude, was slow and unfeasible for studies that require a large sample size, which most cohort studies often do. Recruitment through both of these methods was considerably improved when the advertisement: 1) was posted by a collaborating charity with an existing social media following or 2) used targeted Facebook postings incurring a minimal advertising cost (Facebook ‘boosts’). An audit of the reach of our study advertisements demonstrates that in both the cases, Twitter and Facebook are inefficient sources of identifying cancer survivors: as reported elsewhere too [57, 58], there was very low pull-through recruitment rate from the total population reached, despite high (and increasing) population-level prevalence of cancer. It is interesting that our Twitter recruitment was so much poorer than that reported by Keaver *et al* [28]; however, there are two important differences between that publication and our own work. First, our work recruited directly into a comparatively high-burden research study rather than exploring (in a cross-sectional survey) willingness to participate in future studies. Second, all of their Twitter recruitment were through established collaborator accounts, whereas, here, we attempted (with little success) to recruit using a study-specific Twitter account. In this regard, our data agree with Rabin *et al* [27] that these strategies are probably most effective when partnerships are made with existing organisations with established social media followings. Recruitment through Reddit was reasonably successful, approximately equivalent to that of paid Facebook advertisements, though obviously without the cost implications. In all cases, online recruitment was more effective than either general population community recruitment and a print newspaper advertisement in our study.

In light of these data, we would recommend that a combination of 1) targeted Reddit community postings, 2) fee-payable Facebook ‘boosting’ and 3) recruitment via existing cancer-related Twitter accounts is likely to be the most effective online combination strategy. Due to limited turnover of membership of each of these online communities, we remain unconvinced as to how effective multiple repeated recruitment drives would be over time: our second wave of Twitter posts through charity partners, for example, resulted in far reduced recruitment than the first of these attempts. The total sample size achievable may, therefore, have a ceiling effect. Notwithstanding these limitations, and despite some published evidence to the contrary [15], we have clearly demonstrated the cost-effectiveness of these recruitment methods in comparison to clinic-based recruitment, though this will be tempered by the need, in many cases, for recruitment to be completed over a short time frame than we were able to achieve. As a result, our conclusion is that while appropriate for some studies, recruitment through online social media is certainly not a feasible replacement for more traditional methods of recruiting patients in the clinical setting for all types of cancer survivorship research.

In this study, we recruited a sample of cancer survivors which was more heterogenous than we often otherwise see in psychosocial oncology research. Some research questions might well require a more homogenous sample construction, but where there are no fundamental theoretical or conceptual reasons to do so, limiting the generalisability of findings through non-representative recruitment is problematic [4]. We have demonstrated that where the goal is to maximise representability, online recruitment methods successfully identify younger participants, presumably because social media usage–while increasing in older populations [17]–remains higher in younger age groups. In this context, however, it is understandable why recruitment was slow: cancer diagnosis is far rare in these younger age groups and hence recruitment methods focussed here miss higher prevalence demographic groups. There may well be a case to be made for a recruitment strategy with mixed traditional and online recruitment until generational issues in social media usage become less pronounced. Based on the study by Keaver *et al* [28], we were also not able to demonstrate that online recruitment can overcome the gender bias of participants in cancer survivorship research; this is a considerable problem for our field and there are clearly other reasons why men do not participate in our research than need to be further explored. We were interested to see that we recruited a larger sub-sample of participants who identified as non-heterosexual than in other survivorship research [44, 59]; we suspect that the anonymity of our recruitment and data collection methods may have led to a higher rate of disclosure of non-heterosexuality. This is aligned with systematic review evidence that suggests that online recruitment may be more effective for ‘hard-to-reach’ target populations [15]. Although we recruited from a larger range of geographic regions than we might otherwise have achieved, our sample was still biased towards English-speaking, developed nations; online advertisements and availability of the questionnaire in alternative language formats may have countered this, though there would have been additional study set-up costs in doing so.

One final limitation that we must highlight here is that there may be a confounding effect of multiple online components of this study: online recruitment rates may well have been limited, for example, because participants did not wish to complete data collection online too. Similarly, it is reasonable to assume that the longitudinal nature of our study, and the perceived participation burden inherent in it, may have been off-putting. It is possible that online recruitment may well be more successful with less complex study designs.

## Conclusions

In their own conclusions, Whitaker *et al* [21] and Kapp *et al* [57] suggest that while there is potential for online recruitment in this kind of research study, there is still much to learn about how to optimise the method. We are inclined to agree: the decision to recruit online is complex and while it may overcome cost limitations, and may protect against some sample biases, other biases and unanticipated methodological limitations may also be introduced [58]. Our experiences were somewhat positive, but we by no means recommend this as a panacea to sampling and recruitment issues in cancer survivorship research. Since we initiated recruitment into our study, ethical recommendations for the use of social media as a research recruitment tool have been published [60] and we encourage other researchers considering using these methods to consult these guidelines to build sustainable and ethical research practice.

## Conflicts of interest

The authors declare that they have no conflicts of interest.

## Funding

This project was funded by a research grant from the University of Chester, and supported by a charitable donation from Cancer: A Need for Change.

## Figures and Tables

**Table 1.** Clinical and demographic sample characteristics.

**Gender**	Female (*n* = 170)	85%
	Male (*n* = 30)	15%
**Age**	Mean = 47.5 years (SD = 13.42 years; range = 16–79 years)	
	16–30 years (*n* = 26)	13%
	31–65 years (*n* = 160)	80%
	65 years or over (*n* = 14)	7%
**Relationship status**	Married/De Facto (*n* = 106)	53%
	Single (*n* = 58)	29%
	Divorced/separated (*n*=36)	18%
**Sexual orientation**	Heterosexual (*n* = 174)	87%
	Non-heterosexual + (*n* = 26)	13%
**Location**	Europe (*n* = 106)	53%
	North America (*n* = 72)	36%
	Australia (*n* = 14)	7%
	Other (*n* = 8)	4%
**Cancer**	Breast (*n* = 74)	37%
	Other (*n* = 70)	35%
	Colorectal (*n* = 30)	15%
	Gynaecological (*n* = 26)	13%
**Diagnosis**	Primary (*n* = 148)	74%
	Recurrent (*n* = 54)	26%
**Treatment**	Ongoing (*n* = 120)	60%
	Completed (*n* = 80)	40%

## References

[1] Hulbert-Williams NJ, Beatty L, Dhillon H (2018). Psychological support for patients with cancer: evidence review and suggestions for future research. Curr Opin Support Palliat Care.

[2] van Lankveld JJDM, Fleer J, Schroevers MJ (2018). Recruitment problems in psychosocial oncology research. Psycho-Oncology.

[3] Ransom S, Azzarello LM, McMillan SC (2016). Methodological issues in the recruitment of cancer pain patients and their caregivers. Res Nurs Health.

[4] Howell DC (2013). Fundamental Statistics for the Behavioral Sciences.

[5] McMillan SC, Weitzner MA (2003). Methodologic issues in collecting data from debilitated patients with cancer near the end of life. Oncol Nurs Forum.

[6] Newington L, Metcalfe A (2014). Factors influencing recruitment to research: qualitative study of the experiences and perceptions of research teams. http://www.biomedcentral.com/1471-2288/14/10.

[7] Thewes B, Rietjens JAC, van den Berg SW (2018). One way or another: the opportunities and pitfalls of self-referral and consecutive sampling as recruitment strategies for psycho-oncology intervention trials. Psycho-Oncology.

[8] Watson E, Rose P, Neal R (2012). Personalised cancer follow-up: risk stratification, needs assessment or both?. Brit J Cancer.

[9] Stanton AL, Luecken LJ, MacKinnon DP (2013). Mechanisms in psychosocial interventions for adults living with cancer: opportunity for integration of theory, research and practice. J Cons Clin Psychol.

[10] Patel MX, Doku V, Tennakoon L (2013). Challenges in recruitment of research participants. Adv Psychiat Treat.

[11] White C, Gilshenan K, Hardy J (2008). A survey of the views of palliative care healthcare professionals towards referring cancer patients to participate in randomized controlled trials in palliative care. Support Care Cancer.

[12] McFadden E, Bashir S, Canham S (2015). The impact of registration of clinical trials units: the UK experience. Clin Trials.

[13] Darbyshire JH (2008). The UK clinical research network—building a world-class infrastructure for clinical research. Rheumatol.

[14] NCRI (2013). NCRI clinical studies groups: a prospectus. http://csg.ncri.org.uk/wp-content/uploads/2014/01/2013-NCRI-CSG-prospectus.pdf.

[15] Topolovec-Vranic J, Natarajan K (2016). The use of social media in recruitment for medical research studies: a scoping review. J Med Internet Res.

[16] Wyatt D, Hulbert-Williams NJ, Swash B, Wyatt D, Hulbert-Williams NJ (2015). The context of cancer care. Cancer and Cancer Care.

[17] Zickuhr K, Madden M (2012). Older adults and internet use. https://www.sainetz.at/dokumente/studien/Older_adults_and_internet_use_2012.pdf.

[18] Kelion J (2017). Smartphone sales boom with over-55s. https://www.bbc.co.uk/news/technology-41319684.

[19] Seltzer ED, Stolley MR, Mensah EK (2014). Social networking site usage among childhood cancer survivors—a potential tool for research recruitment?. J Cancer Surviv.

[20] Statista (2019). Facebook: number of monthly active users worldwide 2008–2018. https://www.statista.com/statistics/264810/number-of-monthly-active-facebook-users-worldwide/.

[21] Whitaker C, Stevelink S, Fear N (2017). The use of Facebook in recruiting participants for heath research purposes: a systematic review. J Med Internet Res.

[22] Akard TF, Wray S, Gilmer MJ (2015). Facebook advertisements recruit parents of children with cancer for an online survey of web-based research preferences. Cancer Nurs.

[23] Pederson ER, Kurz J (2016). Using Facebook for health-related research study recruitment and programme delivery. Curr Opin Psychol.

[24] Statista (2019). Total global visitor traffic to Reddit.com. https://www.statista.com/statistics/443332/reddit-monthly-visitors/.

[25] Shatz I (2017). Fast, free and targeted: reddit as a source for recruiting participants online. Soc Sci Comp Rev.

[26] Statista (2019). Twitter: number of monthly active users 2010–2018. https://www.statista.com/statistics/282087/number-of-monthly-active-twitter-users/.

[27] Rabin C, Horowitz S, Marcus B (2013). Recruiting young adult cancer survivors for behavioural research. J Clin Psychol Med Settings.

[28] Keaver L, McGough A, Du M (2019). Potential of using Twitter to recruit cancer survivors and their willingness to participate in nutrition research and web-based interventions: a cross-sectional study. JMIR Cancer.

[29] Hayes SC, Strosahl KD, Wilson KG (2011). Acceptance and Commitment Therapy: The Process and Practice of Mindful Change.

[30] Hulbert-Williams N, Storey L, Wilson K (2015). Psychological interventions for patients with cancer: psychological flexibility and the potential utility of acceptance and commitment therapy. Eur J Cancer Care.

[31] Hulbert-Williams NJ, Storey L (2016). Psychological flexibility correlates with patient-reported outcomes independent of clinical or sociodemographic characteristics. Support Care Cancer.

[32] Swash B, Bramwell R, Hulbert-Williams NJ (2017). Unmet supportive care needs and psychological distress in haematological cancer survivors: the moderating role of psychological flexibility. J Contextual Behav Sci.

[33] Bond FW, Hayes SC, Baer RA (2011). Preliminary psychometric properties of the acceptance and action questionnaire–II: a revised measure of psychological inflexibility and experiential avoidance. Behav Ther.

[34] Gámez W, Chmielewski M, Kotov R (2014). The Brief Experiential Avoidance Questionnaire: development and initial validation. Psychol Assess.

[35] Gillanders DT, Bolderston H, Bond FW (2014). The development and initial validation of the cognitive fusion questionnaire. Behav Ther.

[36] Brown KW, Ryan RM (2003). The benefits of being present: mindfulness and its role in psychological well-being. J Person Soc Psychol.

[37] Trompetter HR, Ten Klooster PM, Schreurs KMG (2013). Measuring values and committed action with the Engaged Living Scale (ELS): psychometric evaluation in a nonclinical sample and a chronic pain sample. Psychol Assess.

[38] Lovibond SH, Lovibond PF (1995). Manual for the Depression Anxiety Stress Scales.

[39] EuroQol Research Foundation (2013). EQ-5D-5L User Guide.

[40] Gotay CC, Pagano IS (2007). Assessment of survivor concerns (ASC): a newly proposed brief questionnaire. Health Qual Life Outcomes.

[41] Carver CS, Antoni MH (2004). Finding benefit in breast cancer during the year after diagnosis predicts better adjustment 5 to 8 years after diagnosis. Health Psychol.

[42] Green SB (1991). How many subjects does it take to do a regression analysis?. Multivar Behav Res.

[43] Wakefield CE, Fardell JE, Doolan EL (2017). Participation in psychosocial oncology and quality-of-life research: a systematic review. Lancet Oncol.

[44] Hulbert-Williams NJ, Plumpton CO, Flowers P (2017). The cancer care experiences of gay, lesbian and bisexual patients: a secondary analysis of data from the UK cancer patient experience survey. Eur J Can Care.

[45] Hulbert-Williams N, Neal R, Morrison V (2012). Anxiety depression and quality of life after cancer diagnosis: what psychosocial variables best predict how patients adjust. Psycho-Oncology.

[46] Dunn J, Ng SK, Holland J (2013). Trajectories of psychological distress after colorectal cancer. Psycho-Oncology.

[47] Robison LL, Mertens AC, Boice JD (2002). Study design and cohort characteristics of the childhood cancer survivor study: a multi-institutional collaborative project. Med Pedatr Oncol.

[48] Lam WW, Shing YT, Bonanno GA (2012). Distress trajectories at the first year diagnosis of breast cancer in relation to 6 years survivorship Psycho-Oncology.

[49] Foster C, Haviland J, Winter J (2016). Pre-surgery depression and confidence to manage problems predict recovery trajectories of health and wellbeing in the first two years following colorectal cancer: results from the CREW cohort study. PLoS One.

[50] Fenlon D, Richardson A, Addington-Hall J (2012). A cohort study of the recovery of health and wellbeing following colorectal cancer (CREW study): protocol paper. BMC Health Serv Res.

[51] Fenlon D, Seymour KC, Okamoto I (2013). Lessons learnt recruiting to a multi-site UK cohort study to explore recovery of health and well-being after colorectal cancer (CREW study). BMC Med Res Methodol.

[52] Woods VD, Montgomery SB, Herring RP (2004). Recruiting black/African American men for research on prostate cancer prevention. Cancer.

[53] Ford JG, Howerton MW, Lai GY (2008). Barriers to recruiting underrepresented populations to cancer clinical trials: a systematic review. Cancer.

[54] Ream E, Quennell A, Fincham L (2008). Supportive care needs of men living with prostate cancer in England: a survey. Brit J Cancer.

[55] Hoyt MA, Rubin LR (2012). Gender representation of cancer patients in medical treatment and psychosocial survivorship research: changes over three decades. Cancer.

[56] Patel G, Harcourt D, Naqvi H (2014). Black and South Asian women’s experiences of breast cancer: a qualitative study. Divers Equal Health Care.

[57] Kapp JM, Peters C, Oliver DP (2013). Research recruitment using Facebook advertising: big potential, big challenges. J Cancer Educ.

[58] Pedersen ER, Helmuth ED, Marshall GN (2015). Using Facebook to recruit young adult veterans: online mental health research. JMIR Res Protoc.

[59] Lisy K, Ward A, Schofield P (2019). Patient-reported outcomes of sexual and gender minority cancer survivors in Australia. Psycho-Oncology.

[60] Gelinas L, Pierce R, Winkler S (2017). Using social media as a research recruitment tool: ethical issues and recommendations. Am J Bioeth.

